# Supplementary material for: Serum Concentrations of Imidazole Dipeptides and Serum Amyloid A in a Bottlenose Dolphin (Tursiops truncatus) with Rhabdomyolysis: Potential Biomarkers for Muscular Damage
Source: Animals (Basel). 2025 Jul 2;15(13):1950. doi: 10.3390/ani15131950 (PMC12249384; doi:10.3390/ani15131950)
Supplement: Supplementary file 1 [file animals-15-01950-s001.zip › Fig_S1.pdf]

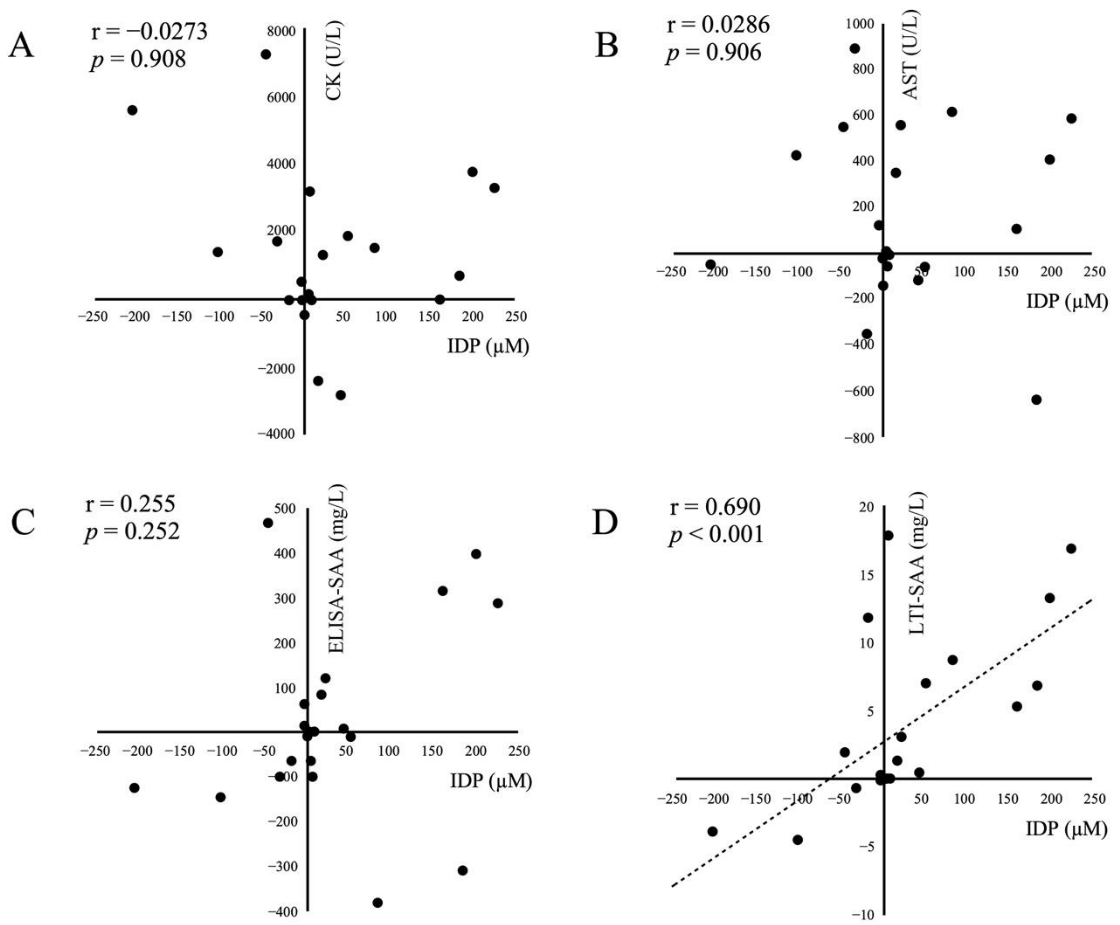

Figure S1. Correlation coefficient (r) and *p* value of differenced series (difference between two points) between total imidazole dipeptides (IDP) and other parameters: A, creatine kinase (CK); B, aspartate aminotransferase (AST); C, serum amyloid A (SAA) measured using an enzyme-linked immunosorbent assay specific to dolphin SAA (ELISA-SAA); and D, SAA concentration measured using a latex turbidimetric immunoassay specific to human SAA (LTI-SAA). Positive (r = 0.690) and significant ($p < 0.001$) correlation was detected between the IDP concentration and LTI-SAA (D), indicating that LTI-SAA concentrations changed in synchronization with the changes in the IDP concentration. However, there was no significant correlation between IDP and the other parameters (A: CK, B: AST, and C: ELISA-SAA). The dots represent the observations and a broken line represents the linear regression model for a significant correlation.
