# Supplementary material for: Serum Concentrations of Imidazole Dipeptides and Serum Amyloid A in a Bottlenose Dolphin (Tursiops truncatus) with Rhabdomyolysis: Potential Biomarkers for Muscular Damage
Source: Animals (Basel). 2025 Jul 2;15(13):1950. doi: 10.3390/ani15131950 (PMC12249384; doi:10.3390/ani15131950)
Supplement: Supplementary file 1 [file animals-15-01950-s001.zip › Table S1.pdf]

Table S1. Concentrations of anserine, carnosine, balenine, and total imidazole dipeptides (IDPs) in the serum of a 5-year-old female bottlenose dolphin with rhabdomyolysis.

Day	−129	−67	−65	−60	−38	−15	1	2
Anserine (µM)	1.61 (4.5)*	1.39 (4.4)	1.40 (4.4)	1.17 (4.2)	1.37 (4.3)	1.70 (4.9)	1.79 (4.9)	2.01 (4.5)
Carnosine (µM)	7.91 (22.3)	7.79 (24.6)	7.57 (24.0)	7.12 (25.4)	7.78 (24.2)	6.69 (19.2)	7.51 (20.4)	8.81 (19.7)
Balenine (µM)	25.98 (73.2)	22.49 (71.0)	22.55 (71.5)	19.74 (70.4)	23.02 (71.6)	26.48 (75.9)	27.59 (74.8)	34.03 (75.9)
Total IDP (µM)	35.50	31.67	31.52	28.03	32.17	34.88	36.89	44.85

Day	3	4	5.5	6	7	7.5	8	9
Anserine (µM)	8.36 (4.1)	6.69 (6.5)	2.56 (3.7)	13.81 (4.7)	13.64 (4.3)	14.72 (4.6)	16.4 (4.8)	18.07 (4.7)
Carnosine (µM)	33.34 (16.2)	25.96 (25.4)	9.28 (13.4)	51.53 (17.4)	34.14 (10.8)	45.78 (14.2)	48.24 (14.2)	45.50 (11.9)
Balenine (µM)	163.82 (79.7)	69.63 (68.1)	57.64 (83.0)	230.04 (77.9)	269.26 (84.9)	262.23 (81.3)	274.22 (80.9)	318.19 (83.3)
Total IDP (µM)	205.52	102.28	69.48	295.38	317.04	322.73	338.86	381.76

Day	9.6	10	10.6	11.5	12	13	14
Anserine (µM)	13.59 (3.7)	27.8 (4.9)	16.03 (4.5)	28.71 (5.3)	22.05 (4.5)	30.10 (5.2)	30.77 (4.9)
Carnosine (µM)	36.70 (10.1)	61.18 (10.9)	35.31 (9.9)	67.19 (12.4)	34.17 (6.9)	55.38 (9.6)	50.25 (8.0)
Balenine (µM)	312.76 (86.1)	474.01 (84.2)	306.05 (85.6)	445.94 (82.3)	439.06 (88.6)	493.19 (85.2)	548.64 (87.1)
Total IDP (µM)	363.05	562.99	357.39	541.84	495.29	578.66	629.67

* Percentage (%) of each IDP among total IDPs.
